# Sodium-Glucose Cotransporter‑2 Inhibitor Improves Renal Injury by Regulating the Redox Profile, Inflammatory Parameters, and Pyroptosis in an Experimental Model of Diabetic Kidney Disease

**DOI:** 10.1021/acsptsci.4c00552

**Published:** 2025-04-16

**Authors:** Paulo Andre Bispo Machado-Junior, Andre Lass, Julia de Bortolo, Leticia Bressan Anizelli, Mateus T. Rocha, Henrique Machado Sousa Proença, Stephanie Rubianne Silva Carvalhal, Samya Hamad Mehanna, Seigo Nagashima, Luiz Claudio Fernandes, Lucia de Noronha, Thyago Proença de Moraes, Ricardo A. Pinho

**Affiliations:** † Laboratory of Exercise Biochemistry in Health, Graduate Program in Health Sciences, 28100 Pontifical Catholic University of Paraná (PUCPR), 1555 Imaculada Conceição Street, Curitiba, Parana 80215-901, Brazil; ‡ Graduate Program in Health Sciences, Pontifical Catholic University of Paraná (PUCPR), 1555 Imaculada Conceição Street. Curitiba, Parana 80215-901, Brazil; § Nephrology Division, 28105 Universidade Federal de São Paulo (UNIFESP), 1500 Sena Madureira, São Paulo, São Paulo 04021-001, Brazil; ∥ Department of Physiology, 28122 Federal University of Parana (UFPR), Curitiba, Parana 81531-970, Brazil; ⊥ Laboratory of Experimental Pathology, Graduate Program of Health Sciences, Pontifical Catholic University of Paraná (PUCPR), 1555 Imaculada Conceição Street, Curitiba, Parana 80215-901, Brazil

**Keywords:** diabetic kidney disease, inflammatory response, NLRP3 inflammasome, sodium-glucose cotransporter-2 inhibitors

## Abstract

Inflammatory response, oxidative stress, and pyroptosis play important roles in the pathogenesis of diabetic kidney disease (DKD), and the NOD-like receptor protein 3 (NLRP3) inflammasome complex and pyroptosis are possible cellular regulators dependent on these processes. Treatment of DKD relies on sodium-glucose cotransporter-2 inhibitors (SGLT2is); however, its effects on oxidative stress and the NLRP3 complex have not yet been fully elucidated. This study aimed to evaluate the role of a SGLT2i in the regulation of the redox system, inflammatory profile, and NLRP3 inflammasome in an experimental model of DKD. Briefly, C57BL/6 mice were subjected to a DKD model induced by the combination of a high-caloric diet and streptozotocin (40 mg/kg). The animals were exposed to empagliflozin 35 mg/kg, and clinical (plasma glucose, water and caloric intake, and weight gain) and functional (glycosuria and albuminuria) parameters were subsequently evaluated. After 25 weeks, the animals were euthanized for evaluation of histological parameters, redox activity, NLRP3 complex activity, and pyroptosis. Our results showed that DKD model animals had clinical features of DKD, namely, high body mass index, glucose levels, albuminuria, and glomerular area. Empagliflozin reduced glycemia levels, glomerular area, H_2_O_2_ levels, IL-1β, IL-1α, and TNF-α levels, lipid peroxidation, and protein carbonylation. It also improved urinary albumin excretion and decreased gasdermin D levels. No changes were observed in the NLRP3 complex proteins. In conclusion, the SGLT2i empagliflozin improved glycemic control and reduced glomerular damage through control of the redox profile and inflammatory parameters, indicating its potential as a treatment for DKD.

## Graphical abstract

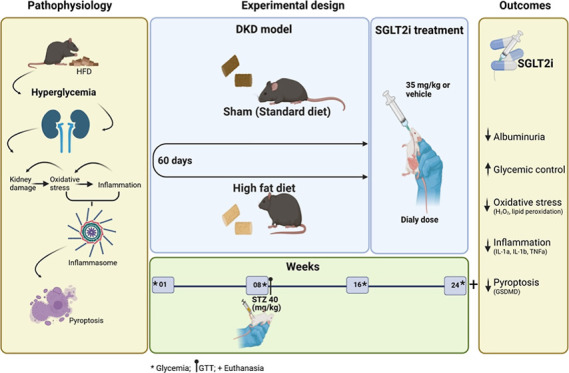

Chronic kidney disease (CKD) is a global public health issue characterized by the progressive and irreversible loss of kidney function.^1^ Diabetes mellitus is considered to be the main cause of CKD worldwide. Approximately 40% of individuals with the disease are expected to develop CKD, which manifests as diabetic kidney disease (DKD), making this disease the main etiology of CKD.^2^

Chronic hyperglycemia is associated with hyperfiltration, leading to an increased glomerular pressure and a subsequent rise in the glomerular filtration rate (GFR) and glomerular hypertension. Over time, this persistent elevation in GFR exerts mechanical stress on the filtration barrier, causing nephron damage, proteinuria, and the development of CKD.^3^ These changes are marked by nodular and/or diffuse glomerulosclerosis, tubulointerstitial fibrosis, mesangial expansion, and basement membrane thickening, all characteristic alterations of DKD (Sharma, McCarthy, Savin, & Srivastava, 2017).^4^

The complexity of CKD highlights the importance of innate immunity,^5^ inflammatory response, and oxidative stress in its development and progression.^6,7^ These effects are attributable to the increase in reactive oxygen species (ROS) and pro-inflammatory cells and cytokines, such as interleukin (IL)-1β, IL-18, and tumor necrosis factor-alpha (TNF-α).^8,9^

Recent studies have highlighted the nucleotide-binding oligomerization domain-like receptor protein 3 (NLRP3 inflammasome), a multiprotein and cytosolic complex, as a major regulator of the inflammatory response in DKD. The inflammasomes are multimeric protein structures, and at least five types of inflammasomes have been identified, with the nucleotide-binding domain, leucine-rich-containing family, pyrin domain-containing-3 (NLRP3)^10^ being the most well characterized. In the context of DKD, modulation of the NLRP3 complex can prevent inflammation and slow the progression of fibrosis,^11^ reducing the consequences of diabetic nephropathy^12^ and protecting against disease progression. Thus, the attenuation of the inflammatory response through modulation of the NLRP3 complex has become a new therapeutic strategy in the context of kidney diseases.

Sodium-glucose cotransporter-2 inhibitors (SGLT2is), a class of oral antidiabetics introduced to the market in 2012 with the goal of slowing disease progression by lowering blood glucose levels through excretion in the urine,^13^ have similarly shown metabolic benefits and the ability to slow DKD progression in several recent clinical studies. SGLT2i acts on the proximal kidney tubules, inhibiting glucose reabsorption independently of insulin or pancreatic beta cell function. As a result, SGLT2i has shown benefits in cardiovascular and kidney outcomes,^14^ primarily owing to improved glycemic control via the regulation of glycated hemoglobin, fasting glucose, postprandial glycemia, blood pressure reduction from urinary sodium loss,^15^ and weight loss.^16^

Recently, SGLT2is have been found to modulate the inflammatory response in different animal models, reducing the progression of kidney disease.^17,18^ SGLT2is reduce pro-inflammatory markers by inhibiting macrophage activity,^19^ suppressing molecular pathways,^20^ and reducing oxidative stress.^21^ SGLT2is inhibit the activation of the NLRP3 inflammasome in several animal models, including in cases of obesity, lung injury, myocardial infarction, diabetic nephropathy, depression, and atherosclerosis.^20,22^

Nevertheless, the impact of SGLT2is on redox parameters and the NLRP3 complex is not yet fully understood owing to the complexity of DKD and the numerous animal models proposed to study the disease.^23^ Understanding the mechanisms involving kidney damage caused by diabetes and proposing potential renoprotective interventions are necessary, given the global importance of the disease. In this sense, we investigated the effects of empagliflozin (35 mg/kg) on clinical and metabolic parameters using a model combining a high-fat diet (HFD) with low-dose streptozotocin-induced (STZ) diabetic mice.

## Materials and Methods

### Animals and Bioethical Procedures

All experiments were conducted according to the standards and ethical principles of the Brazilian College of Animal Experimentation and ARRIVE 2.0, approved by the Ethics Committee for the Use of Animals of the Federal University of Paraná (Protocol number: 23075.018158/2021-09). Eight-week-old male C57BL/6J mice (*n* = 48, weight 20 g) were purchased from Fundação Carlos Chagas (Curitiba, Brazil). The animals were housed in groups of four mice in polypropylene cages at constant temperature (23 °C ± 2) under a 12 h light/dark cycle and with free access to water and food.

### Induction of Type 2 Diabetes Mellitus

Type 2 diabetes mellitus (T2DM) was induced according to the recommendations of the Diabetes Complications Consortium. For this purpose, two complementary methods were used: HFD (60% fat, 20% protein, and 20% carbohydrate) (PRAG Soluçes, Brazil) and a single low-dose of STZ (40 mg/kg). This model was used because of the capacity of STZ to cause damage to β-pancreatic cells, while the HFD in C57BL/6 mice mimics the systemic alterations compatible with T2DM in humans (obesity, hyperglycemia, hypertriglyceridemia, and hypertension).^24^ A high-caloric diet can cause albuminuria, glomerular expansion, and collagen deposition, and the STZ administration helps mimic the natural history of T2DM in humans. Therefore, this model is suitable for investigating the pathophysiology of T2DM complications and testing the effectiveness of possible therapeutic agents.^25,26^

### Experimental Protocol

The animals were assigned to four experimental groups based on their original housing boxes to minimize stress and potential aggressive behavior that could arise from changing their living environment: normal control mice with standard diet (SHAM group, *n* = 12), normal control mice treated with 35 mg/kg of empagliflozin (SHAM + EMPA group, *n* = 12), T2DM mice with HFD and intraperitoneal injection of STZ (DKD group, *n* = 12), and T2DM mice with HFD and intraperitoneal injection of STZ and treatment with 35 mg/kg of empagliflozin (DKD + EMPA group, *n* = 12). The diabetic groups were fed HFD for 8 weeks before receiving a single intraperitoneal (IP) injection of STZ (40 mg/kg, Sigma-Aldrich, St. Louis, MO, USA) dissolved in sodium citrate buffer (10 mM, pH 4.5) after a 6 h fast. After 72 h, blood glucose was measured in caudal vein samples, and animals with levels >180 mg/dL after a 6 h fast were maintained in the study. Following 16 weeks of HFD, empagliflozin treatment (35 mg/kg dissolved in 0.5% hydroxyethylcellulose) was administered daily via oral gavage for 8 weeks, while control groups received vehicle. This dosage of empagliflozin corresponds to the equivalent active dose in humans^27^ and is similar to the dose range used in prior experiments.^28–31^

### Metabolic Data

Water and food intake were measured every 2 d. Food intake was measured by dividing food consumption by the number of animals/cages. The body weight of the mice was measured every 2 weeks using a 0.001 g Filizola precision scale.

The fasting glucose was determined from blood drawn from the tail vein after an overnight fast using a glucometer (On Call Plus II) at 0, 8, 17, and 25 weeks. A glucose tolerance test was performed after a 12 h fast at week 8 by administering glucose IP (2 g/kg body weight), and blood glucose was measured 0, 15, 30, 60, and 120 min postglucose administration.

To collect urine samples, the animals were placed in metabolic cages for 24 h at week 25. Urine was collected and stored at a temperature of −80 °C for further analysis. Urine glucose was determined using commercially available assays (Glucose PAP Liquiform—Labtest), and the urinary albumin level was determined using 10% SDS-polyacrylamide gel electrophoresis. The urine was diluted to a 1:20 concentration and mixed with a buffer solution containing a reducing agent (mercaptoethanol).^32^ The gel was stained with Coomassie blue, and albuminuria was quantified using an Amersham Imager 600 (GE Healthcare Life Sciences).

At the end of the study (week 25), the mice were euthanized by cervical displacement. Samples from the right kidney cortex were directly sectioned into four or five equal portions, which were immediately stored in liquid nitrogen (−140 °C) and then stored in a freezer (−80 °C) for biochemical assays. The left kidney (*n* = 6 per group) was fixed in 10% phosphate-buffered formaldehyde for 24 h, embedded in paraffin blocks, and sectioned into 5 μm sections. The remaining tissue from the animals was packed in a milky white bag, sent to a freezer at the university, and then collected for transport for later deposit in an appropriate landfill.

### Histological Analysis

Histological slides were prepared with hematoxylin–eosin stain for general observations of histopathology with a light microscope. Tubular damage was evaluated based on the degree of interstitial fibrosis and atrophy of the tubules, measured using Sirius Red staining. To analyze glomerular expansion, glomerulosclerosis in the renal cortex, and arteriolar hyalinosis, periodic acid–Schiff staining was used to assess renal injury, as previously described.^33,34^ All histological analyses were performed by two independent pathologists, who were blinded to the experimental groups. The slides were numbered sequentially, and the pathologists assessed each slide without knowledge of the group allocation. Briefly, 50 glomeruli from five regions of the nephron (10 glomeruli/nephron) were evaluated in each 2 animals per slide; all measurements were added to obtain the final values. Each glomerulus in each section was graded from 0 to 4, where 0 represented no lesion, and 1, 2, 3, and 4 represented expansion of the mesangial matrix or sclerosis, involving 25, 25–50, 50–75, or 75% of the area of the glomerular tuft, respectively: 0, normal glomerulus; 1, glomerulus with mesangial expansion; 2, glomerulus with sclerosis in 50% of its area; 3, glomerulus with lesions involving 50–75% of its area; 4, glomerulus with lesions ≥75% of its area. The glomerular area was estimated by using Image Pro Plus software.

### Immunohistochemistry

The left kidney samples were imbedded in paraffin and sectioned at a thickness of 5 μm; sections were mounted on positively charged slides, prepared by immersing them in a solution of silane (3-aminopropyltriethoxysilane; Sigma-Aldrich, São Paulo, SP, Brazil) at 5% in acetone (v/v). After fixation, the slides were cleared in xylene, rehydrated through successive passages in ethanol, and washed in distilled water. Next, endogenous peroxidase blocking was performed with 1.5% hydrogen peroxide (H_2_O_2_) in methanol (v/v) for 20 min. After washing, the samples were subjected to antigen retrieval in 0.01 M citrate buffer (pH 6.0) for 40 min at a temperature of 95–98 °C, and the immunodetection of the proteins of interest was carried out using the following primary antibodies: anti-NALP3 (Affinity Biosciences, BF8029, 1:100), anti-ASC-1 (Elabscience, EAB66894, 1:200), anticaspase-1 (ABclonal, A0964, 1:200), antigasdermin D (ABclonal, A18281, 1:100), and antilipocalin associated with neutrophil gelatinase (NGAL, Abcam, AB23477, 1:1000). The slides were then washed with phosphate-buffered saline (PBS) and incubated with the appropriate biotinylated secondary antibody, either antirabbit IgG or antimouse IgG, followed by a reaction with streptavidin–biotin–peroxidase. After 40 min, the slides were washed with PBS, and the immunodetection was completed using a chromogenic solution containing 0.03% 3,3′-diaminobenzidine (3,3′,4,4′-tetraaminobiphenyl tetrahydrochloride; Dakocytomation, Carpinteria, CA, USA) and 0.3% H_2_O_2_. The results were documented by using a digital camera (Sight DS-5ML1) attached to an optical microscope (Eclipse 50i, Nikon, Melville, NY, USA). For the analysis of each antigen–antibody reaction, 12 images were obtained from the histological sections. Immunodetection of gasdermin D was analyzed using a semiquantitative technique that combined the proportion and intensity of positively immunostained cells. For each protein was used the Allred method^35^ for qualitative analysis where the proportion of positive cells was evaluated as a percentage (0%), 1 (≤1%), 2 (2–10%), 3 (11–33%), 4 (34–66%), and 5 (67–100%), and the staining intensity was classified [0—absent; 1—weak (+); 2—medium (++); 3—strong (+++)]. The analyses were conducted in a single-blind manner by two pathologists.

### Inflammatory Markers

For the evaluation of inflammatory markers, a 20 mg portion of the right kidney was homogenized in 200 μL of RIPA buffer in a 1:10 ratio (w/v) containing protease and phosphatase inhibitors to ensure complete cell lysis. After centrifugation, the supernatant containing the total proteins was collected. Subsequently, the samples were quantified and prepared for analysis using a multiplex kit and read using a Luminex (Millipore Corporation, Billerica, USA). The volume administered to the plate was adjusted to a concentration of 200 μg of protein as determined by the Bradford method, following the manufacturer’s instructions. After the samples were added to the multiplex plate, followed by incubation, washing, and detection of IL-1β, TNF-α, IL-1α, MCP-1, IL-6, and IFNγ, the data were analyzed and expressed in pg/mg of protein.

### Redox Parameter Assays

A spectrophotometer (VersaMax ELISA) was used to evaluate the redox parameters. The Amplex Red kit (Invitrogen, Paisley, UK) was used to measure H_2_O_2_ production, with the reagent 10-acetyl-3,7-dihydroxyphenoxazine reacting in a 1:1 stoichiometry with H_2_O_2_ BTO producing highly fluorescent analyte resorufin. Absorbance was measured at 560 nm. Protein carbonyl content was measured following the method of Colombo et al.^36^ Briefly, tissue homogenates were incubated with 2,4-dinitrophenylhydrazine (DNPH) in 2.5 M HCl for 1 h at room temperature in the dark. The proteins were precipitated with trichloroacetic acid and centrifuged. The pellet was washed three times with ethanol/ethyl acetate (1:1) to remove free DNPH and lipids. The final pellet was dissolved in a 6 M guanidine hydrochloride solution. The carbonyl content was determined by reading the absorbance at 366 nm using a molar extinction coefficient of 22,000 M^–1^ cm^–1^. The malondialdehyde (MDA) content was determined using the MAK085 kit (Sigma-Aldrich) by reacting MDA with thiobarbituric acid to obtain a colorimetric reaction reading of 532 nm. The total protein content was measured in all samples to normalize the results obtained in each assay using an optimized Bradford method. Albumin was used as the protein standard, and linear regression results were used to determine the total protein concentration (mg/mL).

### Statistical Analysis

Data are presented as mean and standard deviation. The Shapiro–Wilk test was used to assess data normality. When one or more groups showed non-normal distribution (*p* < 0.05), data were analyzed using the Kruskal–Wallis test followed by Tukey post hoc test. For normally distributed data, differences between groups were analyzed using two-way analysis of variance (ANOVA) followed by Bonferroni post hoc test for multiple comparisons. Additionally, Student’s *t*-test was used for direct comparisons between the SHAM and DKD groups when data showed normal distribution. Statistical significance was set at *p* < 0.05. All analyses were performed using GraphPad Prism version 8.0.

## Results

### Clinical Parameters

#### Body and Kidney Weights

The groups had similar weights at baseline and hence were considered homogeneous at the beginning of the study. At week 17 (W17, preintervention), the groups treated with HFD weighed more than those that received the standard diet and that at the beginning of the study. These findings showed a significant difference (*p* < 0.001) that was maintained at week 25 (W25, *p* < 0.001) (Figure 1a), indicating successful induction of the obesity model. Despite this, empagliflozin (35 mg/kg) did not cause a significant weight loss at week 25. After 30 weeks, there were no differences in kidney size or weight across the four groups (Figure 1b,c).

**Figure 1:**
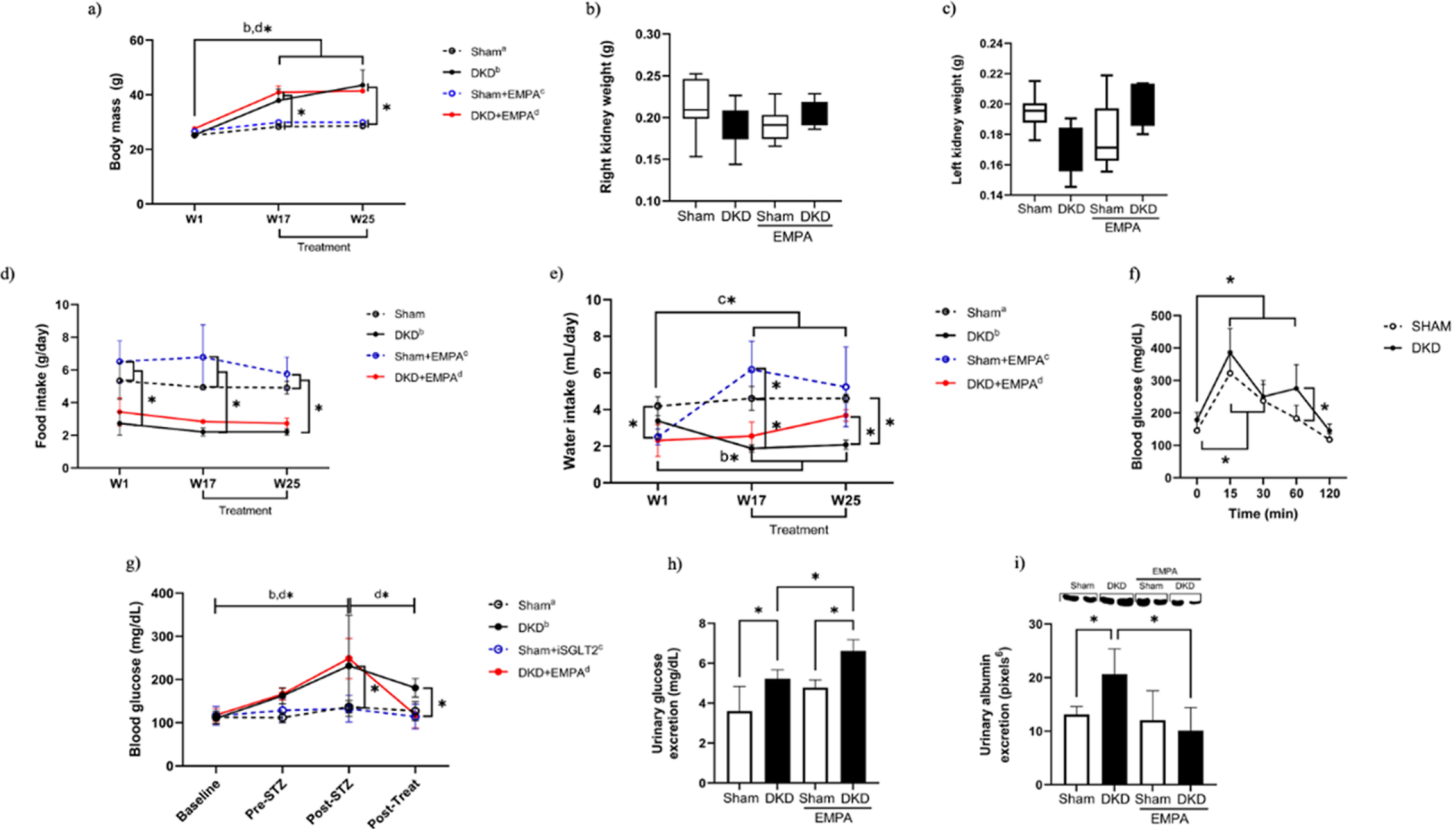
Body mass data (a), right kidney weight (b), left kidney weight (c), food intake (d), water consumption (e), blood glucose levels (f), glucose tolerance test (g), urinary glucose excretion (h), and urinary albumin excretion (i) from animals subjected to an experimental model of DKD. Data are presented as the mean ± standard deviation. Statistical analysis was performed using two-way ANOVA followed by the Bonferroni posthoc test, except for food and water intake which was analyzed using Kruskal–Wallis test followed by Dunńs posthoc test due to non-normal distribution. W1 = week 1; W17 = week 17; W25 = week 25. Sham (*n* = 11), DKD (*n* = 11), Sham + EMPA (*n* = 11), DKD + EMPA (*n* = 10). *p < 0.05.

### Daily Intake of Food and Water

Diabetic groups exhibited a lower average daily food intake compared with nondiabetic groups (*p* < 0.0001), and this difference was sustained throughout the study, with no alterations after administration of empagliflozin. There was no significant variation in caloric intake in the intragroup analyses over the 25 weeks (Figure 1d).

Regarding the water consumption (Figure 1e), the SHAM group showed higher baseline water consumption than the SHAM + EMPA (*p* = 0.004) and DKD + EMPA groups (*p* = 0.002), with no difference when comparing SHAM vs DKD and without significant variation throughout the study. In relation to the DKD group, a decrease in water consumption was observed when comparing week 1 vs week 17 (*p* = 0.01), with no difference when comparing week 1 vs week 25 and lower water consumption at weeks 17 and 25 compared with the SHAM group (*p* < 0.0001). When the DKD and DKD + EMPA groups were compared, a significant difference was observed at the end of the study (*p* = 0.02), with lower water consumption in the DKD group. The SHAM + EMPA group showed an increase in water consumption until week 17 (*p* < 0.0001), and after the initiation of empagliflozin, a nonsignificant decrease was observed (*p* = 0.05).

### Glucose Levels

Glucose levels were similar between the groups at baseline, which demonstrated the homogeneity of the samples. Exposure to the high-caloric diet caused a progressive increase in plasma glucose levels in diabetic animals, with a significant difference observed after STZ administration when comparing the diabetic groups (DKD and DKD + EMPA) versus SHAM (*p* < 0.05). As expected, the use of empagliflozin for 8 weeks decreased glycemia in the treated groups, with a statistically significant difference between the DKD vs DKD + EMPA groups (*p* < 0.05), confirming the effectiveness of the SGLT2 in reducing plasma glucose in diabetic animals (Figure 1g). The glucose tolerance test was performed at week 8 in the SHAM and DKD groups to assess glucose clearance capacity in diabetic animals. The results demonstrate peak glycemia and reduced glucose clearance in the diabetic groups, confirming the effectiveness of the model in inducing glucose intolerance in the animals (Figure 1f).

### Urinary Glucose and Albumin Excretion

A urinary glucose analysis validated the mechanism of action of empagliflozin. The results from week 25 showed that the DKD group exhibited higher levels of urinary glucose than the SHAM group, which was expected given the pathophysiology of DKD. Comparison of the SHAM + EMPA vs DKD + EMPA groups also revealed a significant difference (*p* < 0.05), with increased levels of urinary glucose in diabetic animals exposed to the medication (Figure 1h). A comparison of the DKD vs DKD + EMPA groups revealed a significant increase in glucosuria in the group that received the medication (*p* < 0.05), indicating treatment effectiveness. By week 25, diabetic animals exhibited higher urinary albumin excretion than the SHAM group (*p* < 0.05), demonstrating the presence of glomerular injury. Conversely, animals exposed to empagliflozin showed a significant reduction in urinary albumin excretion compared with diabetic animals, demonstrating the medication’s ability to reduce the degree of renal injury (Figure 1i).

### Redox and Inflammatory Parameters

#### Production of ROS

The data presented in Figure 2a demonstrate the changes in the production of ROS observed between the groups. Diabetic animals exhibited a higher production of ROS (H_2_O_2_) than the other groups, which was reduced by empagliflozin (DKD vs DKD + EMPA groups, *p* < 0.05). The activity of the catalase enzyme was reduced in the DKD + EMPA group than in the SHAM + EMPA group (*p* < 0.05) (Figure 2b).

**Figure 2:**
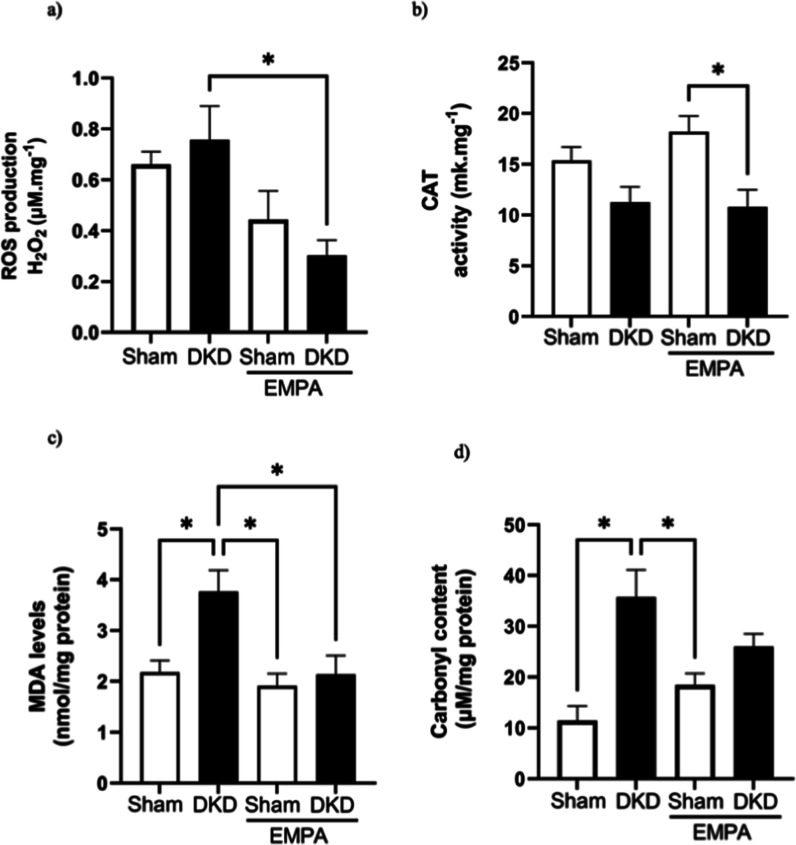
Analysis of ROS production (a), catalase activity (b), levels of lipid peroxidation (c), and carbonylation of proteins (d) from animals subjected to an experimental model of DKD. Data are presented as the mean ± standard deviation. Statistical analysis was performed using two-way ANOVA, followed by the Bonferroni posthoc test, except for MDA production, which was analyzed using the Kruskal–Wallis test, due to non-normal distribution, followed by Dunn’s posthoc test. Sham (*n* = 6), DKD (*n* = 8), Sham + EMPA (*n* = 8), DKD + EMPA (*n* = 6). *p < 0.05.

### Oxidative Damage

To evaluate the oxidative damage, lipid peroxidation (MDA) and protein oxidation (carbonyl levels) parameters were evaluated (Figure 2c,d). Increased levels of MDA were observed in the diabetic group compared with those in the SHAM (*p* = 0.007) and SHAM + EMPA groups (*p* < 0.001), indicating high levels of lipid peroxidation. The DKD + EMPA group had lower levels of MDA than the DKD group (*p* = 0.01), indicating a modulation of oxidative damage with the use of empagliflozin (Figure 2c). Similarly, the diabetic group demonstrated an increase in carbonyl levels compared to the control group (*p* = 0.0004), demonstrating greater protein oxidation and indicating an increase in oxidative damage (Figure 2d). The groups exposed to empagliflozin had reduced carbonyl levels compared to the DKD group (*p* = 0.01).

### Inflammatory Parameters

Levels of pro-inflammatory markers IL-1α (a); IL-1β (b); and TNF-α (f) were significantly increased in the DKD group compared with those in the SHAM group (*p* < 0.05). The administration of empagliflozin significantly reduced these pro-inflammatory cytokine levels in the diabetic group (*p* < 0.05). The levels of MCP-1, IL6, and IFNγ did not differ among the four groups (Figure 3).

**Figure 3:**
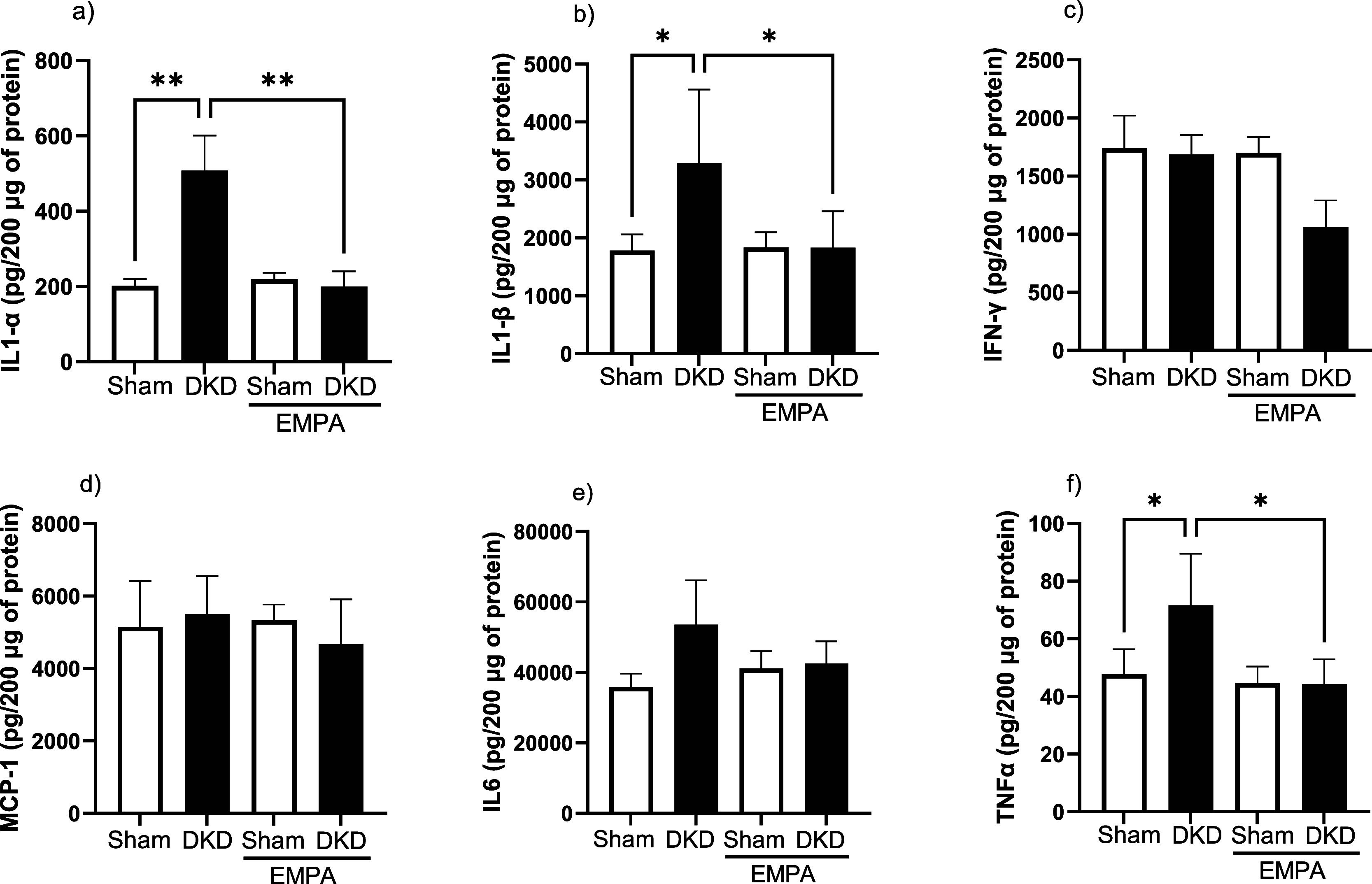
Analysis of the levels of IL-1α (a), IL-1β (b), IFN-Y (c), MCP-1 (d), IL-6 (e), and TNF-α (f) in the right kidney cortex from animals subjected to an experimental model of DKD. Statistical analysis was performed using a two-way ANOVA, followed by the Bonferroni posthoc test. Note that data are expressed by mean ± standard deviation; Sham (*n* = 5), DKD (*n* = 5), Sham + EMPA (*n* = 5), DKD + EMPA (*n* = 5). *p < 0.05; *p < 0.01.

### Renal Histopathology and Immunohistochemistry

#### Glomerular Expansion Area

In Figure 4a, the images related to glomerular expansion assessed by periodic acid Schiff staining were assessed according to the glomerular score ranging from 0 to 4, as described previously. Based on pathologist scoring, the majority of glomeruli in all groups presented with score 0 (normal glomeruli). Among the pathological findings, score 1 (glomeruli with mesangial expansion) was the most prevalent. The percentage of glomeruli with score 1 per group was as follows: Vehicle-Sham (6%), Vehicle-DKD (16.3%), SGLT2i-Sham (6.6%), and SGLT2i-DKD (16.0%). Very few cases (∼3%) reached score 2 (glomerulus with sclerosis in 50% of its area) in DKD groups. Statistical analysis showed no significant differences between any of the groups (*p* > 0.05). The quantitative glomerular scoring data are presented in Table S1. Despite this, a significant increase in the glomerular area (Figure 4b) relative to the total area was observed in the DKD group compared with that in the SHAM group (*p* < 0.0001). This was reduced by empagliflozin (*p* < 0.001). No significant difference was observed between the groups in the evaluation of tubular damage measured using Sirius Red staining (Figure S1).

**Figure 4:**
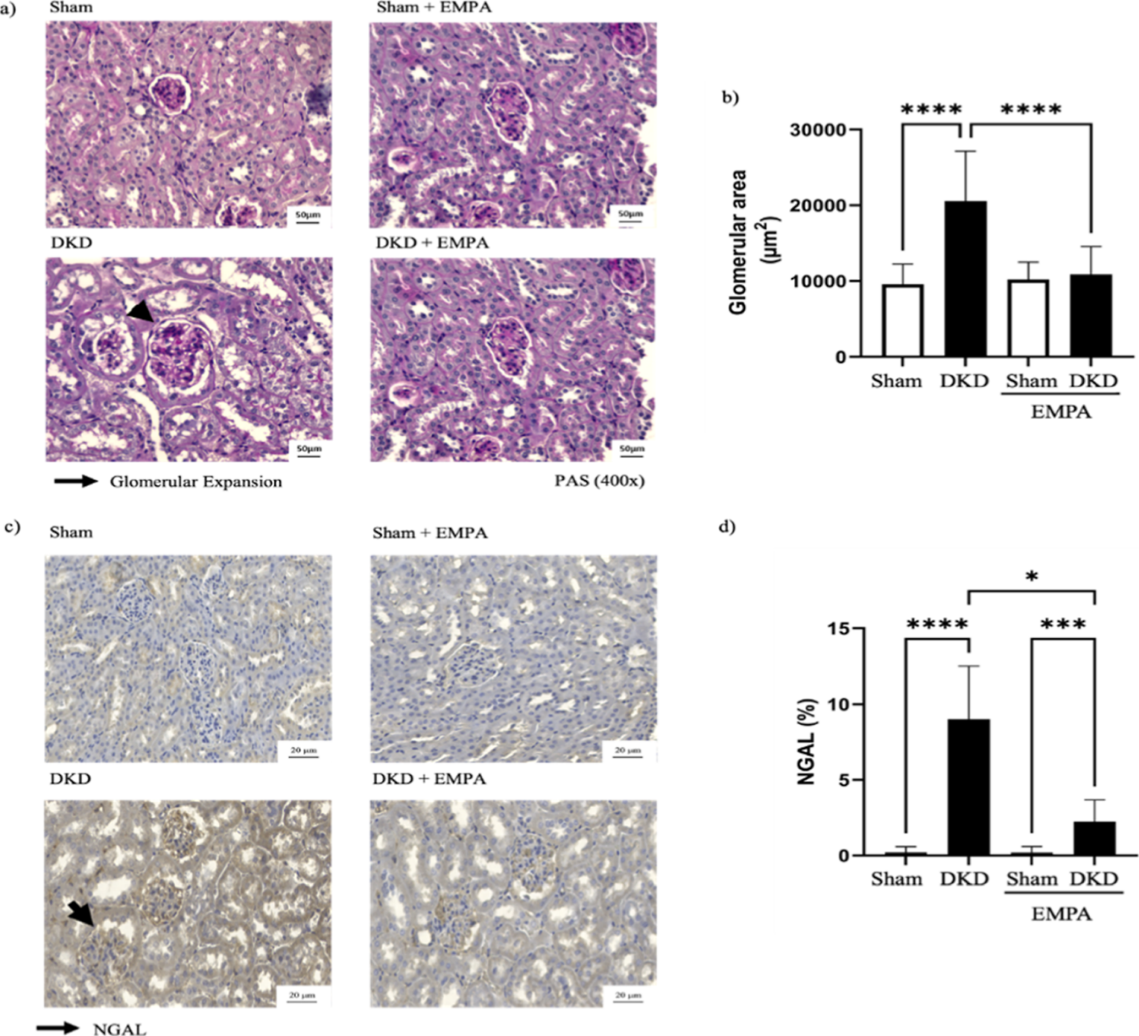
Analysis of glomerular expansion (a), glomerular area (b), and neutrophil gelatinase-associated lipocalin (NGAL) immunoexpression (c and d) in the left kidney of animals with DKD. Representative photomicrographs of periodic acid–Schiff staining (400× magnification, scale bar = 50 μm) showing glomerular expansion (arrow) (a) and NGAL immunostaining (400× magnification, scale bar = 20 μm) with positive staining (arrow) (c). Data are presented as mean ± standard deviation. Statistical analysis was performed using two-way ANOVA, followed by the Bonferroni posthoc test. *p < 0.05, ***p < 0.001, ****p < 0.0001. Sham (*n* = 11), Sham + EMPA (*n* = 11), DKD (*n* = 11), DKD + EMPA (*n* = 10). Inflammasome NLRP3 Activity.

### Anti-Lipocalin Associated with Neutrophil Gelatinase Activity

NGAL is a protein predominantly expressed in the distal part of the nephron after a renal injury. Immunostaining images and NGAL expression are shown in Figure 4c,d, respectively. In the immunohistochemical analysis, a significant increase in NGAL expression was observed in the DKD group compared with that in the SHAM group (*p* < 0.0001), indicating a high degree of renal injury. Administration of empagliflozin reduced the NGAL levels in the DKD group (*p* = 0.03). However, exposure to the drug did not restore NGAL levels to baseline (SHAM + EMPA vs DKD + EMPA, *p* = 0.0005).

Immunohistochemical evaluations were conducted to assess the activity of NLRP3 inflammasome activity (Figure 5a) and pyroptosis (Figure 5b) in kidney samples. The results demonstrated similar levels of NALP3, ASC, and caspase-1 between the groups. Nevertheless, an increase in gasdermin D immunoexpression was observed in the DKD group compared with that in the SHAM group (*p* < 0.0001), indicating a higher degree of pyroptosis. However, the DKD + EMPA group had lower levels of gasdermin D (*p* < 0.0001), indicating a possible mechanism of action. A statistical difference was also observed in the comparison between the SHAM + EMPA and DKD + EMPA groups (*p* < 0.0001). Details of the scoring analyses can be observed in supplementary Table S2.

**Figure 5:**
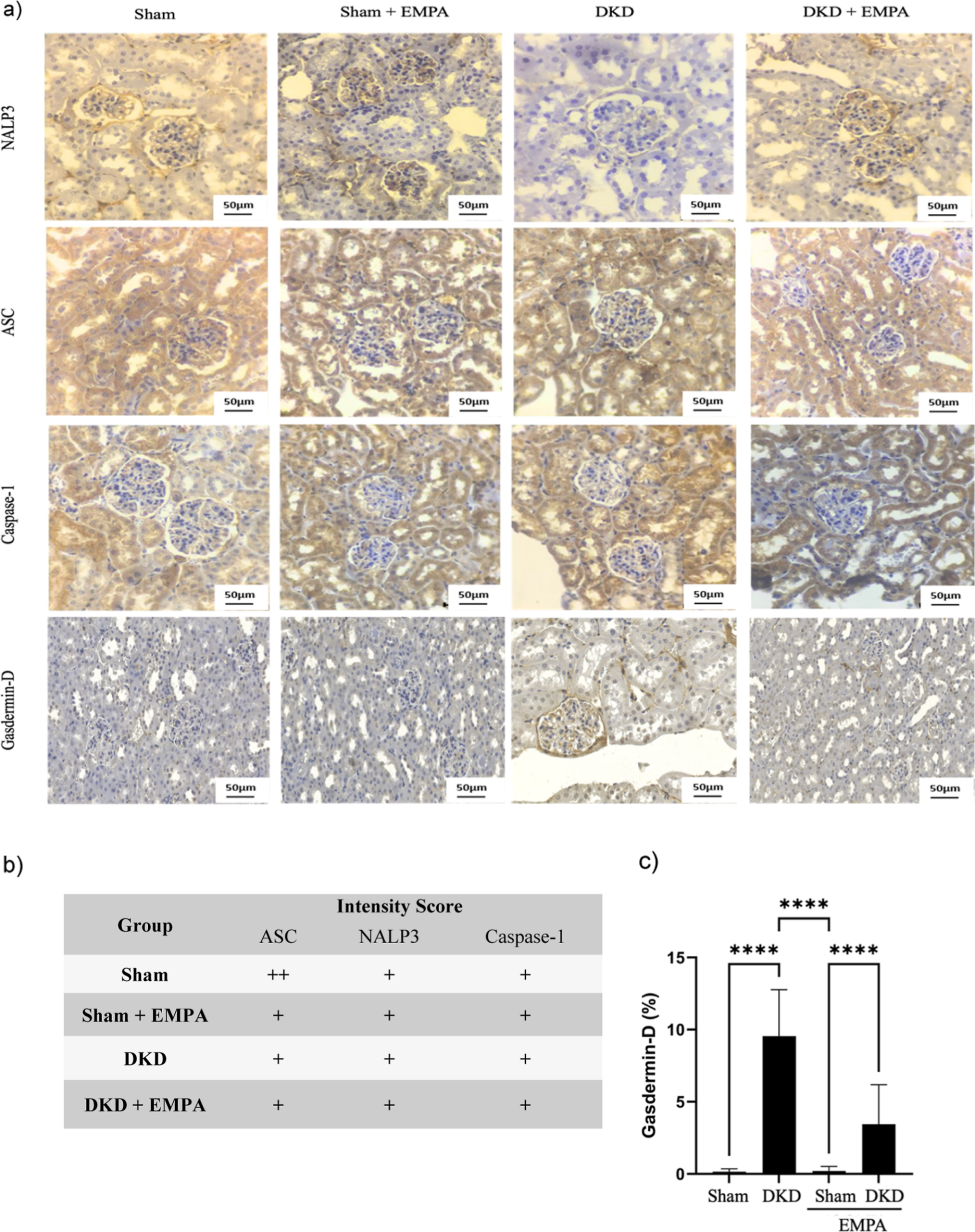
Representative images of immunohistochemical analysis (cross sections) of NALP3, ASC, caspase-1, and gasdermin-D immunostaining (a) (400× magnification, scale bar = 50 μm), classification in intensity score of immunodetection of NALP3, ASC and caspase-1 (b), and immunoexpression of gasdermin D (%) (c), in the kidney of animal models of DKD. For intensity score, + indicates weak staining, ++ indicates moderate staining. Data are presented as mean ± standard deviation. Statistical analysis was performed using two-way ANOVA followed by the Bonferroni posthoc test. ****p < 0.0001. Sham (*n* = 11), DKD (*n* = 11), Sham + EMPA (*n* = 11), DKD + EMPA (*n* = 10).

## Discussion

In the present study, we used the combination of a HFD and low-dose STZ to mimic DKD and demonstrated that empagliflozin (35 mg/kg) promoted positive results in glycemic control and nephroprotective effects through the modulation of oxidative stress and changes in the inflammatory profile. The chronic disease model used was chosen because of its similarity to the human disease, involving the development of obesity and the clinical parameters of metabolic syndrome.^25^ Exposure to a high-caloric diet significantly increased body mass, blood glucose levels, and the degree of urinary albumin excretion compared to animals that received a standard diet. This difference was exacerbated using a low dose of STZ, demonstrating the efficacy of the proposed experimental model. As expected, intervention with an SGLT2 inhibitor significantly reduced the glycemic level in diabetic animals, suggesting the effectiveness of this medication in controlling blood glucose levels.

The DKD group had a higher body mass than the other groups throughout the study, with a statistically significant difference observed at week 25. These results are consistent with those of a previous study.^37^ However, the use of SGLT2i for 8 weeks did not result in a significant weight loss. These results are supported by previous studies that demonstrated similar results in body weight between groups after the administration of SGLT2is, despite the benefits in glycemic levels.^28,30,38,39^ A prior study using dapagliflozin (1 mg/kg) in a similar animal model also showed improvement in glycemic levels but without any changes in animal weight.^38^ In another study, empagliflozin at doses of 10 or 30 mg did not cause significant changes in body weight during 6 weeks of treatment in ZDF rats, despite improvements in blood glucose levels.^40^ Similar to other studies,^41,42^ no significant differences were observed in kidney weights between groups. Although the DKD group showed slightly lower kidney weights, the absence of statistical significance prevents any conclusive interpretation of this parameter. The development of DKD in our model was confirmed by other important parameters of kidney injury, including increased albuminuria and the glomerular area in the DKD group.

Urinary albumin excretion and NGAL levels were assessed to determine the presence of a structural kidney injury. We observed a significant increase in urinary albumin excretion in the DKD group, which may be related to damage to the mechanical barrier and consequent proteinuria in animals with chronic hyperglycemia.^43^ Empagliflozin significantly reduced albuminuria levels, indicating an improvement in the structural damage caused by DKD. This is an important finding because albuminuria is associated with increased cardiovascular events in patients with DKD, which is directly related to the degree of albuminuria.^44^

The DKD model group presented NGAL levels higher than those of the SHAM group, contributing to the characterization and confirmation of experimental diabetic nephropathy. A recent review suggests that NGAL levels can be used as biomarkers for the early detection of DKD, potentially demonstrating an early degree of incipient nephropathy before proteinuria.^45^ The administration of empagliflozin resulted in a significant reduction in NGAL immunoexpression in the DKD group compared with the untreated diabetic group, indicating an improvement in kidney injury. In an earlier study, in mice subjected to a streptozotocin-induced experimental diabetic nephropathy model, urinary NGAL excretion was directly proportional to the degree of urinary albumin excretion, suggesting a relationship in structural damage.^46^

Oxidative stress is crucial for the pathogenesis and progression of DKD,^47–49^ and the generation of ROS can directly mediate glomerular damage.^49^ We observed that the DKD group had higher H_2_O_2_ levels, whereas animals exposed to empagliflozin showed reduced levels compared to the DKD group. We also observed that MDA levels were increased in the DKD group. Elevated lipoperoxidation alters cell membrane fluidity, impairing the activity of enzymes and membrane-associated receptors and leading to cellular dysfunction.^50^ This finding aligns with research by Shao and collaborators, who reported a similar reduction in MDA levels in diabetic animals using the HFD and STZ model of DKD when treated with a high dose of empagliflozin (30 mg/kg) for 8 weeks, although no significant effect was observed with the lower dose (10 mg/kg).^51^

In addition to the positive effects of SGLT2i on MDA levels, the SGLT2i significantly reduced carbonylation levels, an oxidative process that alters cell signaling, regulates pro-inflammatory transcription factors, and causes irreversible protein structural changes.^52,53^ These results highlight the reduction in MDA levels and protein carbonylation, suggesting the potential antioxidant effects of SGLT2is, as previously suggested.^21^ SGLT2i, such as empagliflozin, may exert an indirect antioxidant effect by improving glycemic control and advanced glycation end products, which are both important contributors to elevated oxidative stress and lipid levels.^54,55^ These inhibitors also exhibit anti-inflammatory properties^23,56^ and improve mitochondrial function,^57–59^ thereby further reducing ROS generation.

Pro-inflammatory cytokines also play a fundamental role in the progression of kidney damage in the context of DKD.^60^ High levels of cytokines (e.g., IL-1β and IL-18) have been found in the urine of patients with DKD, which has been related to increased albuminuria, and appears to contribute to the progression of kidney damage.^61^ We report higher levels of pro-inflammatory cytokines (IL-1α, IL-1β, and TNF-α) in the right kidney cortex of mice subjected to the DKD model. The use of SGLT2i for 8 weeks modulated the inflammatory response, resulting in lower renal levels of these cytokines. No differences were observed in MCP1, IL-6, and IFN-γ, consistent with other studies.^61^ Other authors have found similar results, demonstrating good results in reducing pro-inflammatory markers and oxidative stress parameters with the use of SGLT2is in humans, mainly evaluated through serum or urinary levels of markers but without evaluation of kidney tissue.^62^ Using a type 1 diabetes model, Hatanaka et al. demonstrated that dapagliflozin improved the degree of fibrosis, inflammatory response, and oxidative stress in the kidneys.^63^ These results align with an earlier report involving a type 1 diabetes model, which revealed that an SGLT2i (ipragliflozin) significantly and dose-dependently reduced plasma levels of pro-inflammatory markers (IL-6, TNF-α, MCP-1, and CRP).^18^

The discovery of NLRP3 inflammasome as an inflammatory component in the pathogenesis of DKD has led to the development of therapies aimed at inhibiting its activity to attenuate kidney injury.^64^ Despite their importance, few studies have evaluated the role of SGLT2is in modulating the NLRP3 complex in DKD models. Most studies have evaluated the effects of empagliflozin on the NLRP3 complex in animal models of obesity^34,65^ or in the context of genetic manipulation. For example, Benetti et al. assessed the effects of an SGLT2i on the NLRP3 complex in the context of metabolic syndrome in a diet-induced obesity animal model^37^ and discovered, for the first time, that treatment with empagliflozin could improve glycemic levels and pathophysiological changes in metabolic syndrome, in addition to reducing the activation of the NLRP3 complex and IL-1β. Similarly, the use of empagliflozin (10 mg/kg) in mice exposed to a high-caloric diet reduced the activity of IL-1β, NALP3, IL-18, and IL-16, indicating modulation of the inflammasome complex in these animals.^65^

Our results showed no modulation of the canonical NLRP3 inflammasome pathway components (ASC, pro-caspase-1, and NALP3) with the empagliflozin (35 mg/kg) treatment in our model. Similarly, despite improved glycated hemoglobin levels, Gordon et al.^66^ found no impact on NLRP3 complex enzymes when using empagliflozin (10 mg/kg) in T2DM rats. This lack of effect on the canonical pathway suggests that the renoprotective effects of empagliflozin might be mediated through alternative inflammatory mechanisms in DKD. Interestingly, we observed that the DKD group exhibited higher gasdermin D immunoexpression compared to nondiabetic groups, suggesting activation of pyroptotic cell death, and empagliflozin treatment reduced these elevated gasdermin D levels in renal tissue. These findings can be explained by the dual regulation of gasdermin D through both canonical and noncanonical inflammasome pathways. While the canonical pathway involves inflammation sensors that activate caspase-1 through ASC or NLRC adaptors, the noncanonical pathway is activated by caspase-11 (caspase-4/5 in humans).^67,68^ Although these activation pathways are different, they share a common downstream mechanism where activated caspases cleave gasdermin D and release its N-terminal domain, which form membrane pores, ultimately leading to pyroptotic cell death.^68^ Therefore, the observed changes in gasdermin D levels without corresponding alterations in canonical NLRP3 pathway components suggest possible activation through the non-canonical pathway in our DKD model.

The lack of association between empagliflozin and the NLRP3 inflammasome in the context of DKD suggests that either the immunomodulatory action of empagliflozin occurs through other mechanisms that do not necessarily involve the canonical pathway of the NLRP3 inflammasome or the NLRP3 complex was not activated in the model used in the present study. In addition, most studies that evaluated the potential relationship between SGLT2is and the NLRP3 inflammasome used genetically modified models for T2DM or models of obesity induced solely by a HFD, in contrast with the model used in our study.

In conclusion, in our experimental model of DKD, the empagliflozin (35 mg/kg) treatment reduced blood glucose levels, decreased urinary albumin excretion, and attenuated oxidative stress markers and inflammatory parameters in renal tissue. The observed renoprotective effects occurred without significant changes in the canonical pathway components of the NLRP3 inflammasome complex, suggesting the possible involvement of alternative inflammatory mechanisms in this experimental setting.

### Final Considerations

To our knowledge, this is the first study to evaluate the effects of the SGLT2i empagliflozin at a dose of 35 mg/kg for 8 weeks on clinical and redox parameters, inflammatory levels, and the NLRP3 inflammasome pathway in a mouse model of DKD induced by a combination of a HFD and low-dose STZ. Our findings provide data into the mechanisms by which empagliflozin may protect against DKD. Nevertheless, this study has some limitations. First, we did not use a genetically modified animal model, which is different from other studies, which may limit the ability to explore specific genetic influences on empagliflozin metabolism. Second, renal function was not evaluated through creatinine levels but mainly through urinary albumin levels and glomerular damage. Third, translating findings from mice to humans may be challenging due to differences in drug metabolism, immune response, and physiology. Mice often metabolize drugs faster and have distinct immune systems, and models may not fully mimic human diseases, which limits the findings and may not fully replicate the drug effects under human conditions. Additionally, human patients typically present with various comorbidities and concurrent medications, factors not addressed in our controlled experimental setting. Finally, the intervention lasted only 8 weeks, so we cannot determine the long-term effects of empagliflozin.

## Supplementary Material



## Data Availability

All data sets generated and analyzed during the current study are available from the corresponding author on reasonable request.

## Notes

Glomerular scoring and qualitative analysis of immunoreactivity and tubular damage scoring (PDF)
