# Supplementary material for: Sodium-Glucose Cotransporter‑2 Inhibitor Improves Renal Injury by Regulating the Redox Profile, Inflammatory Parameters, and Pyroptosis in an Experimental Model of Diabetic Kidney Disease
Source: ACS Pharmacol Transl Sci. 2025 Apr 16;8(5):1270–81. doi: 10.1021/acsptsci.4c00552 (PMC12070321; doi:10.1021/acsptsci.4c00552)
Supplement: Supplementary file 1 [file pt4c00552_si_001.pdf]

Supporting Information

Sodium-glucose cotransporter-2 inhibitor improves renal injury by regulating redox profile, inflammatory parameters, and pyroptosis in an experimental model of diabetic kidney disease

Paulo Andre Bispo Machado-Junior [1,2], Prof. Andre Lass [1,2], Julia de Bortolo [1], Leticia Bressan Anizelli [1], Mateus T. Rocha [1], Henrique Machado Sousa Proença [3], Stephanie Rubianne Silva Carvalhal [4], Samya Hamad Mehanna [2], Seigo Nagashima [5], Luiz Claudio Fernandes [4], Lucia de Noronha [2,5], Thyago Proença de Moraes[2], Ricardo A Pinho *[1,2]

[1] Laboratory of Exercise Biochemistry in Health, Graduate Program in Health Sciences, Pontifical Catholic University of Paraná (PUCPR), 1555 Imaculada Conceição Street, Curitiba, Parana, Brazil, Zip Code 80215-901.

[2] Graduate Program in Health Sciences, Pontifical Catholic University of Paraná (PUCPR), 1555 Imaculada Conceição Street. Curitiba, Parana, Brazil, Zip Code 80215-901.

[3] Nephrology Division, Universidade Federal de São Paulo (UNIFESP), 1500 Sena Madureira, São Paulo, São Paulo, Brazil Zip Code 04021-001.

[4] Department of Physiology, Federal University of Parana (UFPR), Curitiba, Parana, Brazil, Zip Code 81531-970.

[5] Laboratory of Experimental Pathology, Graduate Program of Health Sciences, Pontifical Catholic University of Paraná (PUCPR), 1555 Imaculada Conceição Street, Curitiba, Parana, Brazil, Zip Code 80215-901.

Corresponding author:
Ricardo A. Pinho
ricardo.pinho@pucpr.br

Table S1. Glomerular scoring

Vehicle						SGLT2i					
**Sham**	**Sham**	**Sham**	**DKD**	**DKD**	**DKD**	**Sham**	**Sham**	**Sham**	**DKD**	**DKD**	**DKD**
**0**	**0**	**0**	**0**	**0**	**0**	**0**	**0**	**0**	**1**	**1**	**0**
**0**	**0**	**0**	**0**	**0**	**0**	**0**	**0**	**0**	**0**	**0**	**1**
**0**	**0**	**0**	**0**	**0**	**1**	**0**	**0**	**0**	**0**	**0**	**0**
**0**	**0**	**0**	**0**	**1**	**0**	**0**	**0**	**1**	**2**	**0**	**0**
**0**	**0**	**0**	**1**	**0**	**0**	**0**	**1**	**0**	**0**	**1**	**0**
**0**	**0**	**0**	**0**	**1**	**0**	**0**	**0**	**0**	**1**	**0**	**0**
**0**	**0**	**0**	**0**	**0**	**0**	**0**	**0**	**0**	**0**	**0**	**0**
**0**	**0**	**0**	**0**	**1**	**1**	**0**	**0**	**0**	**0**	**0**	**1**
**0**	**0**	**0**	**0**	**0**	**0**	**0**	**0**	**0**	**0**	**0**	**2**
**0**	**0**	**0**	**1**	**0**	**0**	**0**	**0**	**0**	**0**	**0**	**0**
**0**	**0**	**0**	**0**	**0**	**0**	**0**	**0**	**0**	**1**	**0**	**0**
**0**	**0**	**0**	**0**	**1**	**1**	**0**	**0**	**1**	**0**	**1**	**0**
**0**	**0**	**1**	**0**	**0**	**0**	**0**	**0**	**0**	**1**	**0**	**0**
**0**	**0**	**0**	**0**	**0**	**0**	**0**	**0**	**0**	**2**	**0**	**0**
**0**	**0**	**0**	**0**	**0**	**0**	**0**	**0**	**0**	**0**	**0**	**0**
**0**	**0**	**0**	**1**	**0**	**1**	**0**	**0**	**0**	**1**	**0**	**0**
**1**	**0**	**0**	**0**	**1**	**0**	**0**	**0**	**0**	**1**	**0**	**0**
**0**	**0**	**0**	**0**	**0**	**0**	**0**	**0**	**0**	**0**	**0**	**0**
**0**	**0**	**0**	**0**	**0**	**0**	**0**	**0**	**0**	**2**	**0**	**0**
**0**	**0**	**0**	**0**	**0**	**1**	**0**	**0**	**0**	**0**	**0**	**0**
**0**	**0**	**0**	**0**	**0**	**0**	**0**	**0**	**0**	**0**	**0**	**0**
**1**	**0**	**0**	**1**	**1**	**0**	**0**	**0**	**0**	**1**	**0**	**0**
**0**	**0**	**0**	**0**	**0**	**1**	**1**	**0**	**0**	**0**	**2**	**0**
**0**	**0**	**0**	**0**	**0**	**0**	**0**	**1**	**0**	**0**	**0**	**0**
**0**	**0**	**0**	**0**	**0**	**0**	**0**	**0**	**1**	**0**	**1**	**0**
**0**	**0**	**0**	**1**	**1**	**0**	**0**	**0**	**0**	**1**	**0**	**1**
**0**	**0**	**0**	**0**	**1**	**0**	**0**	**0**	**0**	**2**	**0**	**0**
**1**	**0**	**0**	**0**	**1**	**0**	**0**	**0**	**0**	**0**	**0**	**0**
**0**	**1**	**0**	**0**	**0**	**0**	**0**	**0**	**0**	**0**	**0**	**0**
**0**	**0**	**0**	**0**	**1**	**0**	**0**	**0**	**0**	**0**	**0**	**0**
**0**	**0**	**0**	**0**	**0**	**0**	**0**	**1**	**0**	**2**	**0**	**0**
**0**	**0**	**0**	**0**	**0**	**0**	**0**	**0**	**0**	**0**	**1**	**0**
**0**	**0**	**0**	**1**	**1**	**0**	**0**	**0**	**0**	**0**	**0**	**0**
**0**	**0**	**0**	**0**	**0**	**0**	**0**	**0**	**0**	**1**	**0**	**0**
**0**	**0**	**0**	**0**	**0**	**0**	**0**	**0**	**0**	**0**	**0**	**0**
**0**	**0**	**0**	**0**	**2**	**0**	**0**	**0**	**0**	**0**	**0**	**0**
**0**	**0**	**1**	**0**	**0**	**1**	**0**	**0**	**0**	**0**	**1**	**2**
**0**	**0**	**0**	**0**	**0**	**0**	**0**	**0**	**1**	**0**	**0**	**0**
**0**	**0**	**0**	**1**	**0**	**0**	**0**	**0**	**0**	**0**	**0**	**0**
**0**	**0**	**0**	**0**	**0**	**0**	**0**	**0**	**0**	**0**	**0**	**0**
**0**	**0**	**0**	**0**	**0**	**0**	**0**	**0**	**0**	**1**	**0**	**0**
**0**	**0**	**1**	**0**	**0**	**0**	**0**	**0**	**0**	**1**	**1**	**0**
**0**	**0**	**0**	**0**	**0**	**0**	**0**	**1**	**1**	**0**	**0**	**1**
**0**	**0**	**0**	**0**	**2**	**0**	**0**	**0**	**0**	**0**	**0**	**0**
**0**	**1**	**0**	**0**	**0**	**0**	**0**	**0**	**0**	**0**	**0**	**0**
**0**	**0**	**0**	**0**	**0**	**0**	**0**	**0**	**0**	**0**	**0**	**1**
**0**	**0**	**0**	**0**	**0**	**0**	**0**	**0**	**0**	**0**	**1**	**0**
**0**	**0**	**0**	**0**	**1**	**0**	**0**	**0**	**0**	**0**	**0**	**0**
**0**	**0**	**1**	**0**	**0**	**0**	**0**	**0**	**0**	**0**	**0**	**0**
**0**	**0**	**0**	**0**	**0**	**0**	**0**	**0**	**0**	**0**	**0**	**0**

Fifty glomeruli from five regions of the nephron (10 glomeruli/nephron) were evaluated in each 2 animals per slide; all measurements were added to obtain the final values. Each glomerulus in each section was graded from 0 to 4, where 0 represented no lesion, and 1, 2, 3, and 4 represented expansion of the mesangial matrix or sclerosis, involving 25, 25–50, 50–75, or 75% of the area of the glomerular tuft, respectively: 0, normal glomerulus; 1, glomerulus with mesangial expansion; 2, glomerulus with sclerosis in 50% of its area; 3, glomerulus with lesions involving 50–75% of its area; 4, glomerulus with lesions ≥75% of its area.

Table S2. Qualitative analysis of immunoreactivity

	NALP		ASC		CASP-1	
	Glomeruli		Glomeruli		Glomeruli	
	Proportion	Intensity	Proportion	Intensity	Proportion	Intensity
DKD	34-66	1	34-66	1	34-66	1
DKD	34-66	1	34-66	1/2	34-66	1
DKD	34-66	1	34-66	1/2	67-100	1
DKD	10-33	1	34-66	1/2	34-66	1
DKD	10-33	1	34-66	1	34-66	1
DKD	2-10	1	34-66	1	67-100	1
SHAM	34-66	1	67-100	2	34-66	1
SHAM	67-100	1	67-100	2	34-66	1
SHAM	34-66	1	67-100	1/2	34-66	1
SHAM	10-33	1	67-100	1/2	34-66	1
SHAM	10-33	1	67-100	1	34-66	1
SHAM	10-33	1	67-100	1/2	34-66	1
SHAM + EMPA	34-66	1	33-66	1	34-66	1
SHAM + EMPA	34-66	1	34-66	1	34-66	1
SHAM + EMPA	34-66	1	34-66	1	34-66	1
SHAM + EMPA	10-33	1	10-33	1	10-33	1
SHAM + EMPA	10-33	1	10-33	1	10-33	1
SHAM + EMPA	34-66	1	67-100	1	67-100	1
DKD + EMPA	10-33	1	33-66	1	34-66	1
DKD + EMPA	2-10	1	2-10	1	2-10	1
DKD + EMPA	34-66	1	34-66	1	34-66	1
DKD + EMPA	34-66	1	34-66	1	34-66	1
DKD + EMPA	2-10	1	2-10	1	2-10	1
DKD + EMPA	2-10	1	2-10	1	2-10	1

Qualitative analysis of inflammasome components immunoreactivity in renal glomeruli. The table presents the distribution of staining patterns and intensity classification of NALP3 (NACHT, LRR and PYD domains-containing protein 3), ASC (Apoptosis-associated Speck-like protein containing a CARD), caspase-1 (Cysteine-dependent aspartate-directed protease-1) immunodetection in four experimental groups: DKD (Diabetic Kidney Disease), SHAM (surgical control), SHAM + EMPA (surgical control treated with empagliflozin), and DKD + EMPA (Diabetic Kidney Disease treated with empagliflozin). For each protein was used the Allred method [35] for qualitative analysis where the proportion of positive cells was evaluated as a percentage (0%), 1 (≤1%), 2 (2-10%), 3 (11-33%), 4 (34-66%), 5 (67-100%) and the staining intensity was classified (0 – Absent; 1- Weak (+); 2 – Medium (++); 3- Strong (+++).

Figure S1. Tubular damage scoring

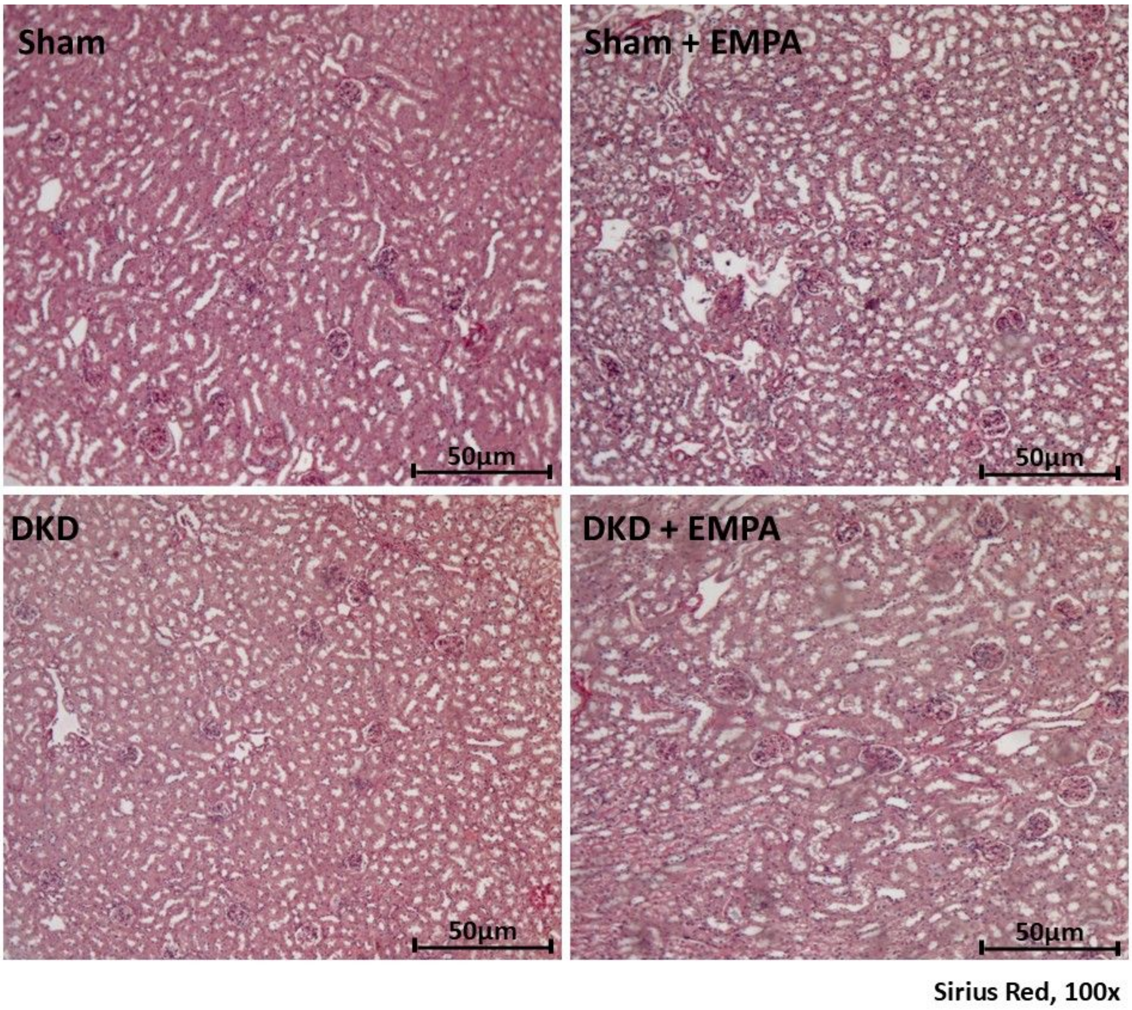

Tubular damage was evaluated based on the degree of interstitial fibrosis and atrophy of the tubules, measured using Sirius Red staining. No significant difference was observed between the groups in the evaluation of tubular damage.
